# Metabolic improvement after exercise training in children with obesity: Possible role of the six-minute walking test

**DOI:** 10.1371/journal.pone.0320209

**Published:** 2025-03-28

**Authors:** Luca Giovanelli, Giuseppina Bernardelli, Simone Facchetti, Mara Malacarne, Matteo Vandoni, Vittoria Carnevale Pellino, Gianvincenzo Zuccotti, Valeria Calcaterra, Daniela Lucini

**Affiliations:** 1 BIOMETRA Department, University of Milan, Milan, Italy; 2 IRCCS Istituto Auxologico Italiano, Exercise Medicine Unit, Milan, Italy; 3 DISCCO Department, University of Milan, Milan, Italy; 4 Laboratory of Adapted Motor Activity (LAMA), Department of Public Health, Experimental Medicine and Forensic Science, University of Pavia, Pavia, Italy; 5 Department of Biomedical and Clinical Science, University of Milan, Milano, Italy; 6 Pediatric Department, Buzzi Children’s Hospital, Milano, Italy; 7 Pediatric and Adolescent Unit, Department of Internal Medicine, University of Pavia, Pavia, Italy; Muhimbili University of Health and Allied Sciences School of Medicine, TANZANIA, UNITED REPUBLIC OF

## Abstract

The aims of this study are to evaluate the effectiveness of an online supervised training program in modulating lipid and glucose metabolism in children with obesity and to investigate the possible role of the 6-minute walking test (6MWT) as a predictor of metabolic improvement. A total of 35 Caucasian children with obesity (aged 8-13) were enrolled in the study and tested before (T0) and after (T1) a 12-week online supervised exercise training protocol: cardiovascular fitness (by means of 6MWT), metabolic biochemical profile, lifestyle (with ad hoc questionnaires focusing on physical activity, nutrition, sedentariness, sleep hours and quality, health perception) and Cardiac Autonomic Regulation (CAR) were assessed. Spearman correlations between the variations in the studied outcomes were explored. After intervention, the distance covered during 6MWT significantly increased (p < 0.001), and nutrition quality improved slightly but significantly (p = 0.03). The improvement in the 6MWT performance was shown to be significantly correlatee with the reduction of insulin levels (r = -0.455; p = 0.02), HOMA-IR Index (r = -0.452; p = 0.02), total cholesterol values (r = -0.549; p = 0.004) and Atherogenic Index of Plasma (AIP) (r = 0.422; p = 0.04). Moreover, there was a significant correlation between the improvement in 6MWT and health perception (r = 0.578; p = 0.002). We observed that the improvement in the 6MWT performance correlates with better metabolic profile after exercise training in children with obesity suggesting the goodness of this simple test on unveil changes in pathogenetic processes underlying obesity.

## Introduction

Childhood obesity is nowadays a huge public health issue worldwide [1]. If such an epidemic is not tackled, the current generation of children may have a shorter lifespan than their parents [2,3]. Notably, children with obesity present higher values of waist circumference, blood pressure and resting pulse rate than normal-weight peers, along with alterations in lipid and glucose metabolism, which may be present before the clear onset of cardiometabolic diseases, such as diabetes or hypertension, and these impairments are associated with an increased cardiovascular risk [3]. In addition to immunological and hormonal mechanisms, there is compelling evidence supporting the key role of dysfunctional autonomic nervous system (ANS) in this context [4–7].

Strategies for preventing and treating pediatric obesity are complex and not exhaustively defined [3]. Actually, physical activity is extremely important for physical, psychological, cognitive and social well-being throughout life, by exerting a positive impact on a wide range of health indicators (e.g., cardiometabolic biomarkers, bone quality, adiposity) [8–10]. However, intervention programs based on physical exercise can be considered effective when – not considering a possible weight loss - they are able to modulate the pathogenetic processes underlying the obesity complications [11]. Although home-based exercises might not be as effective as school- and group-based activities [12], they are undoubtedly more sustainable from both an economical and organizational perspective, and - intriguingly – could serve as an excellent starting point for those children who do not feel like exercising in public. In this regard, COVID-19 pandemic may have represented a catalyst, as people were forced to stay indoors, highlighting the need to redefine the training, testing and monitoring methods [13]. For this reason, it is crucial to verify the efficacy of an online exercise program, to make sure that the intervention drives to improvement in pathogenetic processes underlying diseases, and it is not limited to enjoyment, particularly in childhood.

Multiple tests are currently available for assessing the efficacy of exercise programs. Among these, the Cardiopulmonary Exercise Test is considered as the benchmark for evaluating patient’s fitness, but it is expensive and challenging to administer to young children [14]. On the other hand, Six-Minute Walking Test (6MWT) is quick and easy to perform, cost-effective, widely understood, well-accepted and tolerated, thus potentially becoming a valid and sustainable option [15,16].

The present study aimed to use a relatively simple test, such as the 6MWT, in a group of children with obesity to evaluate the effectiveness of an online supervised training program in modulating lipid and glucose metabolism, whose impairment characterizes obesity even in childhood.

## Materials and methods

Forty Caucasian children (14 females and 26 males), consecutively referred for obesity to the outpatient clinic of Pediatric Unit - Buzzi Children’s Hospital, were enrolled in February 2023. These individuals were assessed before (T0) and after (T1) a 12-week online exercise training protocol, specifically from 8 February to 8 May 2023. Participants fulfilling the following criteria were included: age between 8 and 13 years, body mass index (BMI) z-score ≥  2 (according to World Health Organization’s guidelines) [17]. We excluded those with known secondary causes of obesity, chronic cardiovascular and respiratory diseases, orthopedic problems, absolute contraindications to physical activity, or those who did not understand Italian language. The study received approval from the institutional ethics committee (Milano Area 1, protocol number 2020/ST/298, approved on 2 December 2020) and was conducted in line with the Helsinki Declaration of 1975, as revised in 2008. All participants or their responsible guardians gave their written consent after being fully informed about the study.

Table 1 reports abbreviations and acronyms.

**Table 1:** Description of the variables in the study.

Label	Description
T0	Before intervention
T1	After intervention
Δ	Difference between T1 and T0
6MWT	6 Minutes Walking Test
METs	Metabolic Equivalents
MetsTot	Total Weekly physical activity volume
Sedent	self-reported number of hours per week of sedentariness
AHA.s	American Heart Association Nutrition Score
Health.qual	scale of perceived quality of overall health (in integer scores from 0 to 10)
Sleep.hours	self-reported number of hours per day of sleep
Sleep.qual	scale of perceived quality of sleep (in integer scores from 0 to 10)
SAP.pc	percentile of systolic arterial pressure
BMI.zs	Body Mass Index z-score
WHtR	waist-to-height ratio
FBG	Fasting Blood Glucose
Insul	Insulin
HOMA-IR	Homeostasis Model Assessment – Insulin Resistance
Tot Chol	Total Cholesterol
HDL-C	HDL Cholesterol
LDL-C	LDL Cholesterol
TG	triglycerides
AIP	Atherogenic Index of Plasma
ANS	autonomic nervous system
RR	RR interval
RRTP	RR Total power (RR interval variance)

Every participant underwent the following clinical evaluations: weight, height, waist circumference (WC), pubertal stage as per Marshall and Tanner’s guidelines [18,19], body mass index (BMI), waist-to-height ratio (WHtR) [20]. Evaluations’ details are reported in our previous papers [13,21]. Besides, a basal musculoskeletal evaluation was performed to rule out any musculoskeletal restrictions that could interfere with the exercise program. Systolic arterial pressure (SAP) and diastolic arterial pressure (DAP) were recorded twice with the patient in a supine position after a 5-minute rest. An electronic mercury sphygmomanometer (A & D Medical, Tokyo, Japan) with a suitably sized cuff was used on the right arm [22]. The blood pressure percentile was determined for each child according to the recent guidelines [23,24]. Additionally, every participant underwent a basic electrocardiogram and echocardiogram to rule out significant cardiac diseases that could contraindicate or limit high intensity exercise training.

Regarding the assessment of metabolic biochemical parameters, a blood specimen was collected between 8:30 and 9:00 am (following an overnight fast), and fasting blood glucose (FBG), total cholesterol (Tot Chol), high-density lipoprotein (HDL-C) cholesterol, triglycerides (TG), and insulin (insul) levels were measured, using conventional techniques (Advia XPT, Siemens Healthcare). To evaluate insulin resistance (IR), we calculated HOMA-IR Index as follows: (insulin x glucose)/22,5 [25]. We calculated TG/HDL-C ratio, LDL-C/HDL-C ratio, and Atherogenic Index of Plasma (AIP) according to the formula log(TG/HDL-C), as novel markers for assessing the risk of atherogenicity and cardiometabolic health [26].

In order to assess lifestyle, ad hoc questionnaires were used. In particular:

- nutrition was evaluated by means of the American Heart Association Healthy Diet Score (AHA score), taking into account consumption of whole grain, sodium, sweetened beverages, fish, fruit and vegetables [27], and here considered as an index of nutrition quality;
- hours of sedentariness per week and hours of sleep per day were inquired, as well as perception of sleep quality, health and academic performance, using an evaluation scale from 0 (worst quality) to 10 (best quality) for each measure;
- physical activity (total activity volume) was assessed using a modified version of the commonly employed short version of International Physical Activity Questionnaire (IPAQ) [28,29], which focuses on intensity (nominally estimated in Metabolic Equivalents -METs- according to the type of activity) and duration (in minutes) of physical activity. We decided to employ this questionnaire, even if designed for adults, because it has the advantage of providing a numeric parameter of exercise volume (expressed in METs) which reflects the total exercise volume. We considered the following levels: brisk walking ( ≈ 3.3 METs), other activities of moderate intensity ( ≈ 4.0 METs) and activities of vigorous intensity ( ≈ 8.0 METs). Total Weekly physical activity volume (METs TOT) [MET·minutes/week] =  (3.3 x minutes of brisk walking x days of brisk walking) +  (4.0 x minutes of other moderate intensity activity x days of other moderate intensity activities) +  (8.0 x minutes of vigorous intensity activity x days of vigorous intensity activity).

Participants were tested for cardiovascular fitness at T0 and T1 by means of Six-minute walking test (6MWT) [30,31]. The test was administrated in a straight corridor with a flat surface. Each participant continuously walked at a self-selected pace for six minutes along a 20-m line, with cones placed at each end of the lane. Evaluators showed the test rules before the beginning. To ensure that children understood the instructions, the evaluator performed with the children one trial of one-track length. During the test, every child was observed by a ‘safety chaser’ who gave limited standardized supports [32]. If needed, children were allowed to temporarily stop during the test. The distance covered during the 6MWT was registered in meters. Two kinesiologists received the same training during 4 specific sessions dedicated to the standardization of the test procedures and then supervised the 6MWT procedures. Additionally, test-retest reliability was previously assessed, resulting in a high intraclass correlation coefficient of 0.94 (95% confidence interval: 0.89–0.96) [33].

Cardiac Autonomic Regulation (CAR) was assessed (details on the methodology employed were reported in our previous paper) [13]. Briefly, data were collected on a PC at a rate of 250 samples per second using a custom software tool (HeartScope), which automatically provided a series of indices describing heart rate variability (HRV) in the time domain: RR interval (in milliseconds) and RR interval variability (RRTP) (assessed as total power, i.e., variance, in milliseconds squared), taken as simple classifiers of vagal control [34–36]. Several indices were also provided in the frequency domain (such indices are not here reported because the results of related analysis in this specific population were already published elsewhere) [13].

Based on all the clinical parameters, a medical exercise prescription was created for each participant, defining the type, intensity, duration, frequency, and progression of exercise.

The online exercise training program lasted 12 weeks. The training regimen was structured as three 60-minute sessions weekly for 12 weeks (for a total of 36 sessions), including a variety of exercise types and intensities. Every session was supervised by two sport specialists, and live-streamed via the Zoom platform (California, USA), thus facilitating real-time interaction between instructors and children. Exercises were tailored to the individual fitness levels, and progressively adjusted. More specifically, each session was subdivided into three parts: a preliminary warm-up lasting around 5 minutes to properly prepare children, the main training segment combining aerobic and muscular routines for about 50 minutes, and a final cool-down period of around 5 minutes to ensure bodies return to a rest state. Exercises were all fun and playful. Intensities were gradually increased to moderate and high levels, with maximum heart rates between 50% and 80%, as determined by the Tanaka equation [37]. Short yoga and mindfulness sessions were suggested for cooling down [38]. In order to adhere to the guidelines [10,39] recommending 60 minutes of moderate to vigorous exercise daily for children, we created a dedicated YouTube channel, “LAMA Junior”, which provided adapted exercise routines for days with no supervised training [40].

### Statistical analysis

Descriptive statistics of the studied variables were computed as median ±  MAD (Median Absolute Deviation). Wilcoxon-Mann–Whitney test was used to assess differences between T0 and T1. Spearman correlations were also employed. Statistical analysis was performed using SPSS version 29 (IBM Corp., Armonk, NY, USA). P values < 0.05 were considered statistically significant.

## Results

Thirty-five Caucasian children (14 females and 21 males) completed the study and were assessed both at T0 and T1. Five children did not complete the study for personal reasons.

Table 2 reports the studied outcomes before and after intervention. Some of these variations (anthropometric and 6MWT data) were already shown on a slightly larger study sample in a previous paper our group [21], but the relationship between the changes in 6MWT and other parameters has never been analysed.

**Table 2:** Summary of descriptive data of the study population.

Indices	T0	T1	WMW test p-value
	Median ± MAD	Median ± MAD	
Weight [kg]	62.7 ± 11.4	64.7 ± 9.8	0.003
Height [cm]	152.2 ± 8.3	155.0 ± 7.8	<0.001
6MWT [m]	471.0 ± 38.0	538.5 ± 36.0	<0.001
METsTot [.]	921 ± 652	1395 ± 480	0.027
Sedent [.]	60.0 ± 8.0	62.5 ± 16.0	0.5
AHA.s [.]	2.0 ± 1.0	2.0 ± 1.0	0.03
Health.qual [.]	7.5 ± 1.5	8.0 ± 1.0	0.39
Sleep.hours [h/day]	8 ± 1	9 ± 1	0.25
Sleep.qual [.]	9 ± 1	9 ± 1	0.54
SAP.pc	80 ± 15	66 ± 21	0.73
BMI.zs [SD]	2.10 ± 0.28	1.97 ± 0.40	0.003
WHtR [.]	0.59 ± 0.03	0.58 ± 0.02	<0.001
FBG [mg/dl]	89 ± 3	90 ± 5	0.19
Insul [mU/L]	17.4 ± 6.4	16.0 ± 7.2	0.57
HOMA-IR [.]	3.87 ± 1.62	3.78 ± 1.76	0.79
Tot Chol [mg/dl]	162.5 ± 22.5	155.0 ± 18.0	0.07
HDL-C [mg/dl]	46.0 ± 5.0	47.5 ± 4.5	0.38
TG [mg/dl]	101 ± 31	94 ± 30	0.97
AIP [.]	0.39 ± 0.20	0.36 ± 0.16	0.90
TG/HDL-C	2.46 ± 1.36	2.30 ± 0.84	0.75
LDL-C/HDL-C	2.22 ± 0.39	1.86 ± 0.32	0.047
RR [msec]	718.89 ± 65.37	706.03 ± 68.45	0.97
RRTP [msec2]	2070.68 ± 1267.83	1974.04 ± 1092.01	0.83

Data are expressed as median value ±  mean absolute deviation (MAD).

Abbreviations: T0 =  before intervention; T1 =  after intervention; 6MWT =  6 Minutes Walking Test; METsTot =  Total Weekly physical activity volume; Sedent =  self-reported number of hours per week of sedentariness; AHA.s =  American Heart Association Nutrition Score; Health.qual =  scale of perceived quality of overall health; sleep.hours =  self-reported number of hours per day of sleep; sleep.qual =  scale of perceived quality of sleep; SAPpc =  percentile of systolic arterial pressure; BMI.zs =  Body Mass Index z-score; WHtR =  waist-to-height ratio; FBG =  Fasting Blood Glucose; Insul =  insulin; HOMA-IR =  Homeostasis Model Assessment – Insulin Resistance; Tot Chol =  Total Cholesterol; HDL-C =  HDL Cholesterol; TG =  triglycerides; AIP =  Atherogenic Index of Plasma; LDL-C =  LDL Cholesterol; RR = RR interval; RRTP =  RR Total power (RR interval variance).

Wilcoxon-Mann-Whitney (WMW) test. Significant p-values at the 0.05 level are written in bold.

As expected, the distance covered during 6MWT significantly increased after intervention (from 471 ± 38 to 538.5 ± 36 m, p < 0.001). Notably, the values of BMI z-score were significantly reduced, while the total volume of physical activity was significantly increased at T1 as compared to T0. AHA diet score (index of nutrition quality) was slightly but significantly increased after training (respectively 1.89 ± 0.9 and 2.2 ± 1.1, expressed as Mean ±  SD). Regarding the novel markers of atherogenicity and cardiometabolic risk, LDL-C/HDL-C ratio significantly decreased after intervention.

**Fig 1:**
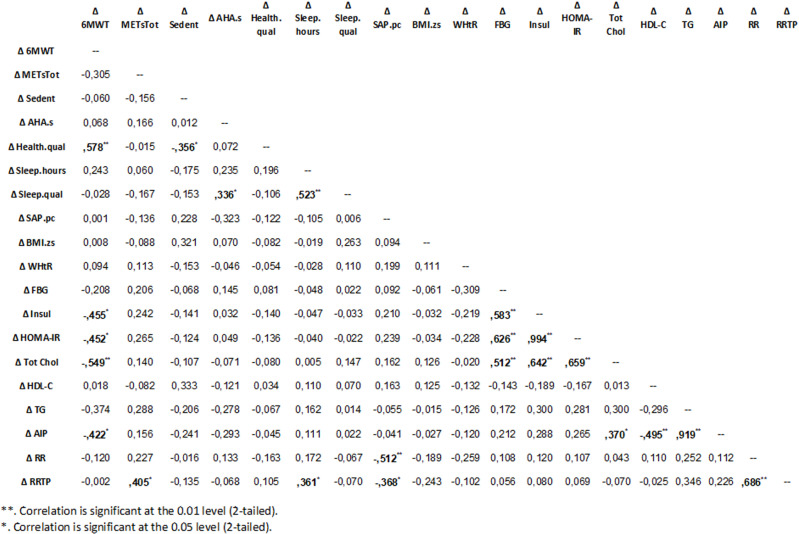
Simple correlation matrix of variations in 6MWT, autonomic, metabolic and lifestyle parameters. Abbreviations: 6MWT =  6 Minutes Walking Test; METsTot =  Total Weekly physical activity volume; Sedent =  self-reported number of hours per week of sedentariness; AHA.s =  American Heart Association Nutrition Score; Health.qual =  scale of perceived quality of overall health; sleep.hours =  self-reported number of hours per day of sleep; sleep.qual =  scale of perceived quality of sleep; SAPpc =  percentile of systolic arterial pressure; BMI.zs =  Body Mass Index z-score; WHtR =  waist-to-height ratio; FBG =  Fasting Blood Glucose; Insul =  insulin; HOMA-IR =  Homeostasis Model Assessment – Insulin Resistance; Tot Chol =  Total Cholesterol; HDL-C =  HDL Cholesterol; TG =  triglycerides; AIP =  Atherogenic Index of Plasma; RR = RR interval; RRTP =  RR Total power (RR interval variance).

We explored the possible correlations between the variations in 6MWT, metabolic, autonomic and lifestyle parameters after the 12-week online supervised training program described above. Fig 1 depicts with a simple Spearman correlation matrix such correlations.

Firstly, the improvement in the distance covered during 6MWT was shown to significantly correlate with the reduction of insulin levels (r = -0.455; p = 0.02), HOMA-IR Index (r = -0.452; p = 0.02), total cholesterol values (r = -0.549; p = 0.004) and AIP (r = 0.422; p = 0.04), Besides, there was a significant correlation between the improvement in 6MWT and health perception (r = 0.578; p = 0.002). The improvement of health perception, in turn, was found to inversely correlate with sedentariness (r = -0.356; p = 0.039).

Moreover, as expected, we found a significant correlation between changes in total cholesterol and changes in other metabolic parameters (insulin, HOMA-IR Index and FBG).

We also observed a significant correlation between the improvement in perceived sleep quality and improvement in AHA nutrition score (a proxy of nutrition quality) (r = 0.336; p = 0.049). Instead, the increase in the hours of sleep correlated significantly with the improvement in RRTP (r = 0.361; p = 0.039), a parameter considered to be an overall marker of vagal control [34–36].

## Discussion

In this paper, we observed that changes in the 6MWT performance correlates with metabolic profile improvements after exercise training in children with obesity.

Specifically, we found that changes in 6MWT correlated with improvements of metabolic parameters, such as insulin, cholesterol, HOMA index and AIP, suggesting that this simple test is able to reveal relevant modifications in the pathogenetic mechanisms underlying obesity-related complications.

Exercise training is considered a strategy of paramount importance to manage obesity and to improve health and well-being in children. In this context, it might be valuable to have a simple test that could be used - also outside the medical setting - to monitor the effectiveness of interventions and to assess even small improvements in the mechanisms underlying obesity. Multiple tests are currently available to assess the effectiveness of exercise programs. Among these, the Cardiopulmonary Exercise Test is considered as the benchmark for evaluating patient’s fitness, but it is expensive and challenging to administer to young children [14]. The same may apply to the Stress Exercise Test. Notably, 6MWT is a reliable assessment tool for measuring physical fitness in children, which may represent a valid alternative, that is more sustainable for many reasons: quick and easy to perform (in any location with a 30-meter corridor, even without specific equipment), cost-effective, widely understood, well accepted and tolerated [15,16]. Moreover, lower performance on the 6MWT has been suggested as an additional feature of cardiometabolic risk clustering in youth with obesity [41]. Nevertheless, the 6MWT has different reference values for children from different countries, and factors such as weight, height, gender and age may influence the performance [42].

The 6MWT has been employed in some studies to evaluate the effects of exercise training in adult patients with metabolic conditions [43,44]. For example, the 6MWT performance at baseline and at follow-up was shown to correlate with several cardiovascular risk markers in adults with obesity, but the percentage increase in the walking distance after the intervention was not influenced by the presence of such markers [43]. Another study showed that overweight COPD patients with metabolic syndrome covered a lower distance during 6MWT than those without metabolic syndrome [44].

The 6MWT has also been used in the pediatric population to analyze the role of obesity and metabolic conditions. Indeed, it was found to be suitable for measuring physical function in pediatric patients with obesity [45]. For example, children/adolescents with obesity showed a reduced walking performance [46] along with an increased haemodynamic response [47] as compared to their healthy weight counterparts. Besides, higher total cholesterol and LDL levels were found to be associated with lower walking distance in young boys [48]. In the present paper, we have addressed the possibility of using this simple test to verify the effect of a training program in children with obesity, without limiting the observation to the effects of obesity on basal walking performance. In fact, we found that improvement in 6MWT significantly correlates with improvements in metabolic parameters such as insulin, cholesterol, HOMA Index and AIP, thus suggesting that it might be a simple and suitable tool to monitor the ability of exercise to affect prominent mechanisms implied in the etiopathogenesis of obesity, even in youth.

The role of insulin and insulin resistance that leads to diabetes and cardiovascular risk is well known even in childhood [49]. Lifestyle interventions are considered key tools in the management of obesity, especially at young age. In this study, we employed physical exercise alone, without any significant change in nutrition (children were only asked to maintain a healthy diet). Notably, we observed a small but significant improvement in the nutrition quality, suggesting that becoming physically active may be associated with diet improvement [11]. Interestingly, the improvement in physical fitness also correlated with a reduction of AIP, an atherogenic index, even in the absence of significant dietary intervention. These findings support the observation in the literature that exercise has benefits beyond the simple reduction of fat mass, and per se positively affects processes associated with cardiometabolic risk.

Additionally, in this study the improvement in 6MWT was correlated with the improvement in health perception, suggesting that the greater is the improvement in physical performance the better is the perception of general health quality. This parameter is nowadays considered to be crucial for overall well-being in children as well [50], which is becoming increasingly relevant [51] in the management of young populations, given the critical role of childhood in determining health and social aspects in adulthood.

This study also presents some limitations. Firstly, it is an observational feasibility study, and the sample size was small, hence limiting the interpretation and generalization of the findings. Studies involving larger pediatric cohorts are required to extend and validate these results. Furthermore, we did not have data on immunologic control, psychological and social aspects.

In conclusion, in this paper we observed that a simple test such as the 6MWT may be useful to evaluate the effectiveness of an online supervised training program in modulating lipid and glucose metabolism, in a group of children with obesity.
